# Observations on Mr. Goldson's Pamphlet

**Published:** 1804-10-01

**Authors:** Alexander Herman Macdonald

**Affiliations:** Surgeon in his Majesty's Service


### Observations on Mr. Goldson's Pamphlet; *by* Alexander Herman Macdonald, M. D. *Surgeon in his Majesty's Service. Communicated by* Mr. Ring.

"Vox diversa sonat; populorum est vox tamen una." Martial.

THE appearance of Mr. Goldson's pamphlet at a period when vaccine inoculation seemed to have conquered every opposition, could not but attract the general notice of the public.

Among no class of men was the surprize so much excited, as among the medical profession. They had witnessed the repeated triumphs of vaccine inoculation, and the downfal of those arguments which were once suggested by the sophistry of a Marcus Herz, the ignorance and self-interest of a Vaume, and the ridicule of a Moseley; and therefore, they could be no strangers to the difficulty which would attend the fabrication of new objections. Their surprize, however, soon ceased, when they perceived that the arguments contained in the pamphlet under consideration, were the very same that were made use of by every opponent, in the earlier part of the practice; with this difference, that in the present instance they are supported by cases and supposed facts; whereas, formerly, they could only be looked upon as mere conjectures. The author's address in obviating the objections which were made against his predecessors, is too striking to pass unobserved. He knows there are cases upon record, which have resisted the variolous infection thirty, forty, and even fifty years subsequent to vaccination, and that these have baffled every opposition; he therefore very prudently pretends to believe, that the casual cow-pox possesses the preventive powers; and carries his faith so far as to advise the Vaccine Institution, "to petition Parliament to lend them once more their fostering hand, (I suppose to make an establishment for keeping cows and greasy horses) that the profession might be better enabled to procure matter immediately from the cow. "This," in his opinion, "would be soliciting them to exert a power truly consistent with their constitutional prerogative; and suited to the temperate wisdom of their deliberations as a legislative body." P. 70 and 71.

Whether it was an anxious wish to discover error, and a sincere love of truth, which induced our author to lay his work work before the public, is a question not easily to be determined. If we were allowed to judge from the facts before us, we should be apt to conclude, that he had been actuated by passions far more powerful. For, to see the Discoverer of Vaccine Inoculation neglect him so far, as to take no notice of his letter,* could not but excite some resentment in a man, who was accustomed to be looked up to by medical practitioners; and when the Rev. Mr. Griffin, a gentleman not belonging to the profession, from motives of the purest philanthropy, encroached upon a practice so glaringly neglected by the medical men in his neighbourhood, as to draw upon them the censure that they were "a century behind the whole world in improvement," p. 6, it is very natural to expect, that one of them should be provoked to stand up for the honour of the Faculty, to clear them from the imputation under which they laboured, or at least to give some reasons to the world, feeble as they are, in extenuation of their neglect. The author, however, is far from acknowledging these to have been his motives; and rather endeavours to conceal them, by making strong professions of candour and liberality. "It is far from his wish to spread vain alarms," (p. 62); yet he has taken great care that his advertisements should make a conspicuous figure in the public papers; and that his pamphlet might be better and more generally known, we are publicly informed,† that he has been very liberal in making a gratuitous distribution of it. Keeping Dr. Jenner's words in his remembrance, he has conducted his experiments, "with that calmness and moderation, which should ever accompany a philosophic research;" accordingly, he never has lost the opportunity of exposing his vaccinated children to the variolous contagion; and when this did not succeed, he made them sleep in the same cradle with others who had the small-pox; took the cap from the head of the one, and clapped it upon the head of the other; and the same filthy cap was made use of through the whole of the contagious period of the disease. (p. 31). In no less than three instances, he has carried his calmness and moderation so far as to persuade parents, who had already witnessed the beneficial effects of the vaccine inoculation in their own family, to submit their other children to the inoculation of the small-pox, for no other purpose but

* See p. 44. and 45.

† Med. and Phys. Journal, No. 65, 66.

but to give him an opportunity of infecting those that were vaccinated, if possible. P. 26, 30, and 66.

The calmness and moderation of his reasoning is equally conspicuous. In the very beginning of his introduction he tells us, that " the vaccine inoculation has been spread throughout almost every part of the civilized world, with a rapidity which stands without example in the history of science ;" and no wonder, for a discovery which was " to render the human constitution unsusceptible of a disease so highly contagious, and universally fatal to mankind, as the small-pox, could not fail to attract immediate attention;" but, after all, this " was an event more to be wished for, than expected." And then, to heighten the surprize, and to raise suspicions, in the minds of the uninstructed, he informs us, that all " these important consequences are to succeed a small puncture with a lancet, without producing any material indisposition, and totally free from any risque of danger;" but this, in our author's opinion, " carries with it an air of mystery;" and he is indeed very much suprized, " that it should have been so readily adopted, and carried to such an extent into practice." P. 1 and 2.

It does not seem to be unknown to Mr. Goldson, " that the success of vaccination is often defeated, either by the matter having been taken from a spurious pustule, or by its having suffered a decomposition from a variety of causes ; in consequence of which, in the early stages of its introduction, blunders had been committed by many practioners, who, to use Dr. Jenner's words, took up the lancet without ever having seen the true vaccine pustule. P. 4. Yet, notwithstanding this, whenever he notices these blunders, he takes care to introduce them in such a manner, as to imply some doubts upon the subject. Thus, after informing us, (p. 3) " that many of the vaccinated children were soon afterwards submitted to the influence of the variolous infection without effect," he immediately makes the following assertion. " Several instances, however, of its inefficacy were observed at this period, but all of them," he adds in a very artful manner, " were pronounced to arise from some cause or other, not at all inimical to the regular practice, pursued by the discoverer, and his own immediate friends." This regular practice was not a secret, as is here insinuated; it was publicly known; and every one who did not deem it too great a trouble, might have made himself acquainted with it. If our author had paid a little more attention to it, perhaps he would not have stood stood in need of asking for further investigation." But I must confess myself at a loss to guess whom he alludes to, when he speaks of the immediate friends of the Discoverer. Surely he cannot call those who first began to practice vaccine inoculation, Dr. Jenner's immediate friends; and therefore, the whole of the preceding passage can be viewed in no other light but that of an insinuation, that the promoters of the vaccine inoculation had formed a plot to enforce the practice upon the public against all reason; or, to use his own words, "to deem every failure spurious, and to conceal it," (p. 62), and thus to class them with their antagonists, who have raised plots, and used means, fair or foul, to obtain their object.

Speaking of the medical practitioners in his neighbourhood, Mr. Goldson is very particular in telling us, that, "they could not remain ignorant of the many instances of failure, which occurred in its infancy Neither could they help remarking, what must have been obvious to every attentive observer, the apparent instability of the practice. With every fresh instance of a spurious case, they heard of new instructions, and cautions in respect to taking the matter. These instructions deviated occasionally, from the thirteenth down to the seventh or eighth day; and yet they were told, that on this point depended the whole success of the operation." P. 6 and 7.

Wonderful instructions indeed! But I must confess, I never heard of them before. These, I suppose, made their first appearance in the world, when I was abroad, where it could not be expected that every paltry publication which appeared on that subject in England, should come to my hands. But I have seen many similar instructions in Germany. For no sooner did the practice of vaccine inoculation begin to be adopted, than the barbers, who, it is well known, practice surgery in that country, to the great nuisance of society, took up their lancets, and inoculated what they called the cow-pox. The consequences might easily have been foreseen. Several of the children inoculated by them, caught the small-pox afterwards. The poor barbers, perceiving that their medical reputation was at a stake, endeavoured to get out of the scrape in the best manner they could. Some of them were bold enough to insist upon it, that the children had the cow-pox in the most satisfactory manner; precisely as had been described by Dr. Jenner, and others of their own countrymen who had written upon the subject. Then the public was annoyed with their justifications; which, generally, consisted of of the recital of some of the most prominent symptoms of vaccination, which they copied almost verbatim from the authors who had written on the subject; and I can assure Mr. Goldson, that this was executed in so masterly a manner, as to bid defiance to any which have appeared in this country, upon similar topics. Others again, more conscientious than the rest, contrived to extricate themselves, by making confessions of their little experience; and by pointing out what they fancied, had led them into error. This, in general, was followed by a code of instructions, which varied, perhaps, as much as those that appeared on this side of the water, but which no sensible person paid any attention to; and, I believe, as little regard was paid in England to the instructions Mr. Goldson alludes to. It reflects no great credit on him to bring them forward, either in support of his argument, or as an excuse for the errors he has committed: principally, as at the same time when vaccination was first practised in his neighbourhood, there were instructions to be found in England, which, to the present time, have not varied at all. To convince our author of this, I shall here copy an extract of a letter I received from Dr. Jenner, dated January 23, 1801, not long after the period, when Mr. Goldson had so much cause to complain about "the apparent instability of the practice;" and I earnestly beg of him to compare the instructions it contains, with those which were in the hands of every one, at the time he wrote his pamphlet.

"I shall take the liberty of laying before you certain rules, which, in my opinion, should guide the vaccine inoculator, and I will venture to add, that were we never to deviate, any occurrence out of the ordinary course of the disease would be extremely rare. The third will perhaps explain the principle upon which your matter parted with its original properties.

"First, We must observe, that the pustule go slowly and regularly through its progressive stages of inflammation, vesication, and scabbing; and that the vesication be accompanied with its efflorescence.

"Secondly, That the vaccine fluid be taken from the pustule for the purpose of inoculation at an early period of its formation.

"Thirdly, If, from any peculiarity in the constitution of the patient, or from the state of the virus, a variety should appear in the character of the pustule, that pustule should not be used for further inoculation.

"The necessity of attending to the first of these rules is is obvious: were it neglected, even an exanthematous blush, excited on the arm by the insertion of the virus, might be deemed a sufficient security; and a mere vesicle, quickly forming, and as quickly subsiding, might be considered as the real vaccine pustule, in which we are to place our confidence.

"With regard to the second injunction, I shall observe, that the activity of the virus begins to diminish upon the formation of the efflorescence; therefore, if circumstances will admit, it should not be taken after the eighth day; but the best guide, I think, would be to stop upon the progress of the efflorescence; I do not presume to say, that at this period the virus is effete; certainly it is not; but that it frequently occasions disappointments, my early trials, and those of others, on whose accuracy I can place the greatest confidence, fully evince.

"As a commentary on the third rule, I shall observe, that when we take the vaccine fluid from a pustule, which, in its progress, has materially deviated from its common character, we are very apt to produce a pustule, maintaining that deviation. The texture of the vaccine virus is certainly very delicate, and easily thrown into derangement; so that causes, apparently trifling, are liable to decompose it. I shall mention one to you, by way of illustration. In the early part of my practice, I used frequently to dry the virus *by the fire*, upon threads, glasses, and lancets, but with much caution respecting the degree of heat; yet experience has taught me, that even this procedure frequently occasioned a variation in the progress of the pustule produced by it, as it was apt to commence with a soft creeping scab; which, in some instances, produced at its edges, as it advanced, the perfect vaccine fluid in a ring around it, which fixed a boundary to the extension of the scab. The efflorescence followed, and the constitution was found secure from the small-pox. But, in other instances, the process ended more abruptly, and then of course, the susceptibility of the vaccine virus remained, which was proved by subsequent inoculation.

"I do not find that the vaccine virus undergoes any change, in passing successively from one patient to another for a great space of time. I can answer for its possessing the same properties at the expiration of twenty months, as when it was taken from the cow."

Having given a few specimens of Mr. Goldson's calmness and moderation, I shall next take into consideration, his qualifications as a vaccine inoculator. In endeavouring to exculpate the medical profession in his neighbourhood from the censure they had incurred, he informs us, that the practice was adopted in the autumn of 1800. All this may be perfectly correct; but he does not inform us, that since this time vaccine inoculation has been practiced there so generally, as in other parts of the world. Having lately visited the place of Mr. Goldson's residence, I have had an opportunity of inquiring into the fact; and I find, that, comparatively speaking, vaccination is even now very little practiced there; and when you talk about the cow-pox, you will hear every where the old story, that children will have the small-pox afterwards. Report says, that at the time Mr. Goldson published his pamphlet, which was about four years after the first introduction of vaccination into Portsmouth, Portsea, and Gosport, he had only vaccinated eighty children. Being at the head of his profession, perhaps no other practitioner had inoculated so many. One of the most respectable physicians had inoculated only two; and we may form a just idea of the high estimation in which vaccine inoculation is held by the medical profession in that part of the country, from "the candid and liberal approbation which they unanimously expressed," when the report of Mr. Goldson's cases, with the observations annexed, were read before the quarterly meeting of the Medical Society of Portsmouth, Portsea, and Gosport. When, therefore, he tells us that the cow-pox was known to the medical men in those towns soon after its promulgation, but little practiced, he has been very unsuccessful in clearing them from the charges under which they labour. However, as they are said to have attended to it with a desire to make themselves masters of the subject," (p. 6) it will be worth while to enquire, with what success these labours have been crowned?

P. 9, he says, that "in no instance, he himself has seen any approach to a spurious disease." I believe there are few vaccine inoculators in Great Britain who can say the same. We were indeed very much surprized when we first read that passage; but our surprize was greatly increased when we were afterwards informed by unquestionable authority, that the practitioners in this neighbourhood never take the matter till what they call the ninth day. But as they do not count the day on which the inoculation is performed, what they style the ninth, is in fact the tenth day. Besides, instead of performing their inoculations from arm to arm, they still continue to dry their matter upon lancets; and then use them as occasion requires.

quires. Therefore, instead of never having seen a spurious cow-pox, it may be questioned, whether Mr. Goldson has ever been so fortunate as to see the genuine cow-pox? P. 55. Speaking of the casual cow-pox, he maintains that the bluish cast is the characteristic of the pustule; that in the first vaccination it retains this colour; but that it is not to be distinguished after it has passed once through the human body. In support of this assertion, he quotes Dr. Woodville's authority; and then adds, that it is "a circumstance of some material import; but which has not been sufficiently attended to."

I must beg to inform our author, that he labours under a very great mistake. Medical men have paid particular attention to this point; and their experience has proved, that the bluish cast is not peculiar to the casual pustule; but, that it is frequently observed in the inoculated cow-pox. I have seen it, not only in the early part of my practice, but likewise, after I had been using matter from the same stock for upwards of three years. Dr. Woodville made this assertion in the beginning of his practice; and I am convinced, that long before this time, he must have frequently witnessed the bluish cast in the inoculated cow-pox.

P. 56. When speaking of the similarity of the vaccine to the variolous pustule, he asserts, that the vaccine pustule "does not possess the permanent power of resisting *its own reiterated action.*" I believe some cases are related by Dr. Jenner, of persons being infected a second time with the Cow-pox. But when we can produce a local infection of the small-pox, in a person who has already undergone that disease; surely it cannot be made an objection to the practice, that the same should happen in the cow-pox. But laying this aside, as Mr. Goldson appears to have a turn for making experiment; I would advise him, to try to infect children a second time with the cow-pox; and he will find, that it is not so easy a matter, and that the cow-pox possesses in a much higher degree than the small-pox, "the permanent power of resisting its own reiterated action." I have made the experiment in several instances, and never have been able to produce the disease a second time but once. I have tried it upon myself; for although I have had the small-pox, I once produced the true vaccine pustule upon my hand. Since that time I have reinoculated myself upwards of a hundred times with cow-pock matter; but have been unable to infect myself again.

P. 56. He talks very learnedly upon the origin of the cow-pox; yet, it is quite unknown to him, that Dr. Jenner, or any one else, since his publication, has ever prosecuted the idea. To the names and labours, therefore, of Mr. Tanner, Drs. Loy, Sacco, and La Font, he is an utter stranger. Had Mr. Goldson made himself acquainted with the experiments of these gentlemen, he would have known, that it appears very doubtful still, whether it is requisite that the matter of the grease should pass through the nipple of the cow, to produce the desired effect in the human body.* With regard to the experiments he proposes, of inserting the morbid matter from the horse's heel into the nipple of the milch mare, I dare venture to foretell, that it would produce nothing at all; since the experiments instituted by Professor Vibourg, in the Royal Danish Veterinary School at Copenhagen, have clearly proved that the grease is not infectious in the horse. He took matter of the grease, and endeavoured to infect the heels of sound horses; he tried various means; such as inoculation, rubbing the heels with the matter, after having shaved off the hair; blistering the parts, and then applying compresses of lint, which had been previously soaked in the matter, &c.; but all his trials proved unsuccessful, no infection took place.† We may therefore safely conclude, that Mr. Goldson's proposed experiments would be attended with similar results.

As a proof how well Mr. Goldson is instructed in every particular, respecting vaccination in his own country, he confounds the Royal Jennerian Society with the Vaccine Pock Institution; and, in his answer to Dr. Walker, in the Medical and Physical Journal, he calls the Doctor the principal vaccinator under the directors of an institution to whom the pamphlet was respectfully addressed.

From the above specimens of our author's erudition in vaccine inoculation, we may form our judgment, how far he was qualified to bring before the public the work under consideration.

But

---

* Professor Wollstein of the Veterinary School at Vienna, has informed me, that what the French call *javarre*, is not the *grease*; the latter is an erysipelatous inflammation of the horse's heel; whereas, the former is a *caries* of the bones, belonging to those parts.—It is therefore easily accounted for why the experiments with matter from the javarre has proved unsuccessful in France.

† See Pfaff & Scheele Nordisches Archiv fur Natur und Arzeneiwissenschaft. B 1. St. 3. Seite 569, 570.

But we are now come to the most important part of the subject, viz. the consideration of the author's proofs, in support of his argument, that vaccine inoculation only proves a temporary preventive against the small-pox. These are illustrated by eight cases; in which the small-pox is said to have happened subsequent to vaccination. Of these, however, four only can be considered to have occurred in his own practice; for Case I. was vaccinated by Mr. Paytherus; Case IV. and V. by Mr. Weymouth; and Case VI. by Mr. Rickman.

The matter used for these inoculations was sent to Mr. Rickman, Surgeon of the division of Marines, by the sick and wounded board; and he made use of it upon five marines. From these he took matter for his own patients; and likewise supplied Mr. Goldson and other Practitioners in the neighbourhood. Whether this matter has not been the source of all the mischief still remains doubtful. Mr. Goldson contends, "that it is not to be presumed that a public board, directing experiments to be made upon the subjects in an Infirmary under their controul, would be so inattentive, as to send such as was improper for that purpose," p. 46. There was not the least occasion for laying so much stress upon this; no body will suspect, that they would send spurious matter intentionally. However it is a fact, I believe, not unknown, that about that period, viz, in autumn 1800, the matter which was in circulation in London, was of a very suspicious nature; and that it was even propagated by some eminent practitioners of the metropolis. And if we take Mr. Rickman's own testimony for granted, perhaps little doubt will remain upon the subject; for in conducting his experiments he "soon found, that the matter ran rapidly into a purulent state after the eighth day; of which he advised the board." p. 49. The experienced vaccinator knows well, how rarely this is the case in the true vaccine vesicle. From my own practice and experience, I am induced to believe, that the true vaccine vesicle never goes into suppuration, except when locally irritated. If you inoculate a very young child, that has not the sense of scratching itself, and care is taken by the nurse not to injure the arm, the vesicle will never pass into suppuration. The matter contained in it will, as it were, congeal; and form a crust similar in form to the original vesicle. In the small-pox the case is different; the fluid contained in the vesicle changes from a limpid into a purulent matter. This is the greatest difference which I have been able to discover betwixt the vesicles of the two diseases.

To this testimony I can add, that it is confidently asserted, that the matter which Mr. Rickman has given to several practitioners in Portsmouth and Gosport, has proved ineffectual; as has been demonstrated by subsequent inoculations. For the sake of humanity, I think it a duty incumbent on the gentlemen to whom this has happened, to come publicly forward, and testify the fact.

On a question of such importance as that under consideration, and which, in Mr. Goldson's opinion, "ought as soon as possible to be determined," no circumstance should have been omitted, that could in the least have elucidated the subject. A faithful Narrative or Journal ought to have been given of the Cases, in which the matter sent by the Board was first tried; then some judgment might have been formed, whether the matter had produced the desired effect or not. The same ought to have been observed, in every subsequent case, in which the small-pox was said to have taken place after the cow-pox. To these particulars, however, the Author has paid but little attention.

Not a word is said of the five marines, who were first inoculated with the matter. Two subjects, who were immediately inoculated from these five, were afterwards affected with an eruptive disease, which was pronounced to be the small-pox. Mr. Goldson has been pleased to give the Cases relative to these two subjects a place in the latter end of his pamphlet; although, in my opinion, an accurate delineation of the symptoms which occurred, both when they were supposed to labour under the cow-pox, and afterwards, under the small-pox, would have greatly tended to the elucidation of the subject; and deserved to have been related the first of all. But Mr. Goldson says, he lays no material stress upon these cases, nor wishes to draw any inference from them. (p. 40.) For my own part I must declare, that I am unable to draw any; for the proofs here offered, that the subjects had the cow-pox, are equally unsatisfactory as those of their having had the small-pox. As I consider one case, as it now stands, unconnected with the other cases related by Mr. Goldson, I shall offer a few remarks upon it here.

From one of the five marines, Mr. Rickman inoculated *Clarke* (Case 6th.) on Nov. 4, 1800. Whether this man had the cow-pock or not, it is difficult to determine; for although nothing had been said of the appearance of the cowpox in the five marines, we are told that "there was no apparent reason to suppose that he had not received every benefit from it, as he (Mr. Rickman) noticed no difference in the appearance of the arm, or the symptoms, from any of the former."

On the 18th of November, he (Mr. Rickman) vaccinated a child, Sarah Smith; as one of the punctures was rubbed off very early, he did not think it proper to note this, as a characteristic case." (p. 41.) Mr. Rickman had no right to draw this conclusion; for as he specifies *one* of the punctures, it follows he must have made *more*; those, therefore, that were not rubbed off, would have proved a sufficient preventive, had the matter been good.

This child, however, caught an eruptive disease, which was deemed the small-pox. But Mr. Goldson himself expresses some doubts whether it was the small-pox; for, he "was struck with a peculiarity in their appearance, which was extremely evident on comparing them with a child in the house adjoining. In each the number was nearly the same; yet they were more prominent and forward in the one than the other." (p. 42.) Nothing is said of the child's constitutional symptoms, nor how long this eruption continued. With matter taken from this child, Clarke was inoculated on March 24, 1802. He sickened on April 1, and an eruption took place; but whether this was the small-pox or not, is equally obscure as in the former case. Mr. Goldson saw him as early as "the third or fourth day at farthest," and confesses that the pustules were "more maturated for the time than might be expected. They were likewise remarkably conical, an observation he had made in the child from whom the matter was taken. The arm had at the same time, a very different appearance from the common small-pox arm; as there was an unusual livid appearance in the maturation of the puncture." (p. 43) "The peculiar appearance of the pustules, connected with what he considered an unusual aspect in the arm, induced him to write to Dr. Jenner on the subject, conveying the suspicions he entertained at that time, of its being an anomalous case of varicella." (p. 44.)

It is barely mentioned that Clarke sickened; but we are not told how long this sickness or the pustules continued. The only additional evidence which is given of its being the small-pox, is, that matter was taken from him, which produced evident proofs in several instances. These several instances are not related; perhaps it produced such a small-pox, that every one who saw it would be surprized with its peculiar appearance, accompanied with an unusual aspect in the arm; the eruptions not maturating, and disappearing in six or seven days, as we shall find to have happened in most cases, which Mr. Goldson considered to be the small-pox.

Upon the whole then, from the inaccurate and careless manner in which this case has been described, no inference whatsoever can be drawn.

In the same light I consider Case IV. and V. which occurred to one Mr. Weymouth, a gentleman who makes a very conspicuous figure in the pamphlet, as an antagonist to vaccine inoculation. No case of failure is quoted by our author; but he calls upon the authority of his friend, Mr. Weymouth, who is always ready to second him.

In Case IV. a child was vaccinated by this gentleman, on March 18, 1801; and the easiest and best proof he gives of the child having had the cow-pox, is, "that the arm inflamed, and put on every appearance described by Dr. Jenner." (p. 36.) But notwithstanding this, when the child was inoculated, thirteen months after, with the small-pox, it caught the disease, became feverish, and had several pustules. Unfortunately, however, one pustule only maturated; but from this one he tinged two lancets, and inoculated two children, both of whom had the small-pox in the most satisfactory manner. I have not the least doubt of it; the pamphlet affords us many opportunities of judging what this gentleman means, when he speaks of having the small-pox in the most satisfactory manner.

Case V. is nothing more or less than a local affection of the small-pox after the cow-pox. There was no eruption whatsoever, but the child had a smart fever. It is hardly worth while to dwell upon this case. The fever was evidently produced by the local irritation, and the same would have happened had the child had the small-pox. Instances of similar local infections after the small-pox are upon record without number; and to bring such a case forward, as an instance of small-pox subsequent to vaccination, betrays no great information, either on the part of our author or of Mr. Weymouth. Mr. Goldson pretends not to lay much stress upon them, (p. 35), why then were they published? or, how can he expect that any one else will lay much stress upon them?

As no conclusions can be drawn from the above cases, I shall take no further notice of them.

With regard to the other cases, which we are now going to consider, the same want of accuracy is conspicuous throughout throughout the whole. We have every reason to call in question the purity of the matter which was used; the proofs that the children had the cow-pox, are indeed too vague to be satisfactory; and whatever Mr. Goldson's erudition may be, on other subjects, his knowledge of vaccination amounts to little more than nothing; so that we cannot deem his authority as satisfactory. We must therefore leave it undecided, whether the children have had the cow-pox or not; but as Mr. Goldson has given us ample proofs, that the cases which he considered to be the small-pox, were diseases of a different description, we shall, in the subsequent remarks, direct our chief attention to that part of his pamphlet.

Case 1. is of a child inoculated with the small-pox, three years and three months subsequently to vaccination. The effects of this latter inoculation were as follows: the arms inflamed, suppurated, and an areola was formed round the parts. On the 26th, "The suppuration was manifestly increased, and the areola was become extremely florid and radiated; bearing evident marks of absorption. The child was pale, not warmer than usual, but its pulse were quicker than *they* should have been, or than *they* ever had been before." These febrile symptoms prove nothing, as they may proceed from local irritation.

When Mr. Goldson asked the parents, "whether the child had been ill during the night, or whether they had observed any kind of appearance on the body. They instantly shewed him six or seven eruptions." It appears very strange to me, that Mr. Goldson should have asked this question; for he informs us that three of these pustules were on the child's forehead and temple, and one on his nose. Had these been the true small-pox pustules, I believe they would have been visible enough without asking.

In the evening of the same day, Mr. Goldson was sent for again; and he found the child in "a high degree of fever, his countenance much flushed, and there was a considerable efflorescence on both arms," which Mr. Goldson considered to be "the rash, which is observed in the inoculated small-pox. Two or three eruptions, of the same kind as those seen in the morning, were readily distinguished through the efflorescence."

It is nothing very astonishing, that a fever should take place in consequence of a local irritation; and this might have been the case here. But there cannot be the least doubt, that the fever in this case was greatly aggravated

 by by the anxiety of the parents; for Mr. Goldson informs us, that the friends began to be alarmed, (p. 13); and that he "perceived the anxiety of the parents led them to watch him with an inquisitive eye." (p. 14) Accordingly it so happened, that on the evening when Mr. Goldson was sent for in haste, the child had been seized with a cold shivering, or, as the author expresses it, "with a violent rigor," which frightened the parents so much, that they pampered the child with hot wines and flannel. We need not wonder, therefore, that Mr. Goldson found him in the state above described, with fever, flushed countenance, &c. With regard to the account of the servants, that he had been delirious the preceding night, no dependance whatsoever can be laid upon it. "Thursday 29, Instead of suppurating, the eruptions were covered with a warty scurf," which is three days after their first appearance, and "this encrustation was rubbed off on the following evening.

Mr. Goldson informs us, that when the history of this case reached London, the opinions of Messrs. Ring, Paytherus, Dr. Willan, and the Medical Society in Bolt Court, differed from that of the medical gentlemen in his part of the country. They all agreed that the attempt to excite the small-pox had failed. "They had likewise no doubt, that the same train of symptoms may be excited in persons who have passed through the small-pox, either in the casual manner or by inoculation." (p. 17.)

Mr. Ring, in his letter to Mr. Grant, has entered more fully upon the subject; and given satisfactory proofs that the case in question was not the small-pox; however, it appears, that Mr. Goldson has shut his eyes and ears to conviction, and persists in his former opinion. He particularly notices an observation of Mr. Ring, which, he says, experience does not confirm; viz. that the sudden disappearance of the pustules "is a sufficient proof that it was not the small-pox, *which always continues a longer time.*" In answer to this, our author proceeds thus: "We well know, that in many instances the inoculated small-pox does not maturate, but retires in a few days; although the patient be perfectly secure. And, as I before observed, one of the gentlemen who saw Mr. Grant's child, remarked, that he had very lately inoculated one, where the appearance of eruption was not greater than in that instance." (p. 19.) I am exceedingly sorry I cannot subscribe to this opinion of Mr. Goldson and his friend. Mr. Ring's assertion is perfectly just; the true variolous erup tion tion goes through a regular course, and always continues a longer time; and no dependance can be placed on an eruption which never suppurated, and only lasted three days. Perhaps, at some future period, we may hear a little more of the case Mr. Goldson's friend speaks of; and then the public will be entertained with a marvellous history of a second infection of the small-pox, in the same manner as we now are with our author's cases of small-pox subsequent to vaccination.

(P. 22.) Mr. Goldson "appeals to the candour of the profession, whether the cases of eruption, pimples, &c. arising from inoculation, in persons who have passed thro' the small-pox, were ever known to bear any kind of proportion to what has occurred in variolous inoculation after the cow-pox?"

This question is asked under the prepossession, that these pustules really were the consequence of inoculation. I am far from being of that opinion; neither can I subscribe to the idea, that the eruption in this case was a sympathetic affection of the skin. I look upon it to have been occasioned merely by accidental infection, from the child having scratched itself, as is daily observed to happen in the cow-pox; for Mr. Goldson informs us, (p. 12) that on Friday the 23d, the fourth day after inoculation, he "found the arm of the eldest had been rubbed in the night, and had discharged some lymph on the linen;" and that the inflammation was considerably increased. In short, the child had scratched its arm, and, with the infected nails, produced the few pimples in question. Again, (p. 23) "During the night of Sunday, the inflammation of the arm rapidly increased." This is another proof that some irritation had been applied to the pustule; for in the small-pox, as well as in the cow-pox, the inflammation proceeds gradually and slowly. During that same night the child was very restless; which by the servants was termed delirious. I am moreover inclined to adopt this opinion, on account of the very short duration of these pimples; and of their being so small that even Mr. Goldson, who was looking out for them, could not see them, till they were pointed out to him.

(P. 22) He further ventures "to appeal to the candour of the most zealous promoters of the cow-pox, whether this circumstance is not very rare, if it does ever happen, when inoculation takes place at an early period after vaccination?" Here I am rather at a loss to understand the author's meaning. If he means the small-pox, nothing but but a direct negative can be given. If he alludes to the eruption; in the manner I have above explained it, it may happen in any case; but besides, such a transient eruption may be produced from a sympathetic affection of the skin, even in persons who have had the small-pox, and consequently it may happen in persons who have had the cow-pox. Or, if Mr. Goldson alludes to the inflammation of the arm, and the febrile symptoms, as I am inclined to think he does, from his subsequent remarks, I can answer him in the affirmative. For although, perhaps, no such cases may be found in Dr. Jenner's publication, they are to be met with in the works of others. Has Mr. Goldson forgotten, or has he never heard of the case of a child called Blondeau, at Paris; which happened a little while after vaccination was introduced in France, and which his predecessor, the famous Vaume, laid so much stress upon, as a case of small-pox subsequent to vaccination? I need not repeat it here, for Vaume has taken care that it should be public enough; but I can relate several which occurred in my own practice, and one in particular, which in Germany made as great a noise, as that of Blondeau in France. Among the first children I inoculated, were those of one Börner, a barber-surgeon in Altona. This gentleman, anxious to gain reputation, took matter secretly from his children, at a period too far advanced, and inoculated several others in his neighbourhood. A short time afterwards, some of these children caught the small-pox, and one of them died. The sensation this created, can easily be conceived. The antagonists to the practice were particularly active, as is the case in general; and, to make it appear of greater moment, it was reported that the matter was taken from children whom I inoculated. After I had enquired into all the particulars, I published them in the Altona Mercury; and exposed Mr. Börner's ignorance. This enraged the gentleman so much, that he immediately inoculated his own children with the small-pox; which was about five months after I had inoculated them with the cow-pox. Even at this early period after vaccination, a local infection took place; and one of the children had a slight attack of fever; but no eruption took place. Mr. Börner's subsequent conduct may easily be guessed: he publicly proclaimed that his children had the small-pox; and there were no Mess. Weymouths, or Seeds, or Hills wanting, to support his cause. Their loquacity, however, was soon silenced by better and abler judges. Since that period I have seen several other instances of local infection tion a short time after vaccination. Indeed, most of my reinoculations were performed very early after my patients had the cow-pox. In the early part of my practice I was obliged to do it, for the satisfaction of the public; but latterly I have left it off entirely. The two last children I reinoculated was a few months after vaccination. I took them to a room where six children were confined with the confluent natural small-pox. I inoculated each child with three punctures on both arms; and left them upwards of an hour in the same apartment, to breathe the variolated atmosphere. In both children the punctures took effect; and six beautiful pustules were formed upon their arms. One child complained a little on the evening of the seventh day, and vomited. The parents were inclined to attribute this to the child's having eaten some unripe fruit that day; but whether it was owing to this cause, or to the irritation of the pustules, I cannot decide; for the child was perfectly well the next morning, and on the tenth day, the pustules in both dried up, without any farther consequence.

(P. 24) He says, "If the same had arisen from an accidental infection, no one would have ventured to doubt." Mr. Goldson is perfectly mistaken, for such cases have occurred to Dr. Woodville, and to Dr. Ballhorn and Mr. Stromeyer at Hanover; not three years after vaccination, but only a few days. The antagonists to vaccination proclaimed that it was the small-pox. Dr. Ballhorn and Mr. Stromeyer, however, cleared themselves from this imputation; but, being led astray by theory, or rather a preconceived idea, they looked upon it as a vaccine eruption. I shall soon have an opportunity of taking more particular notice of these cases.

Case II. III. and the two related in the postscript, are cases in which an eruptive disease, considered by Mr. Goldson as small-pox, occurred, in consequence of exposure to the variolous contagion, subsequent to vaccination.

Case II. is of a child four months old when vaccinated; and we are informed, that since that period she has not been prevented from going where the small-pox might have been. However, she withstood the variolous infection, till she was laid in the same cradle with a child labouring under the small-pox, three years and three months subsequent to vaccination.

Case III. is relative to the child, on whom the manœuvre of the nightcap was performed, but without success; it was, however, infected at school, three years after it it had the cow-pox. The two cases in the postscript are said to have resisted the variolous contagion in the same manner for some time. When the children had the supposed small-pox, the child in Case II. had a fever from Thursday till Saturday noon; and on Sunday seven distinct eruptions appeared. Nothing is mentioned of the external character of the pustules; they only lasted five days, and never maturated.

In Case III. there were upwards of a hundred eruptions, several of which were pustular, and already far advanced towards maturation, on the fourth day of the eruption; the first day on which Mr. Goldson saw the child. They dried off as early as the seventh day. It is said, that previous to the eruption, the child was unwell from Wednesday till Saturday, and even that it had a considerable fever. However this fever cannot have been so very considerable, as the mother of the child was not in the least alarmed; and only considered it to arise from cold. The only thing she thought necessary for the child's relief was to keep it in bed, and give it some diluting drink.

In the Case related, p. 66, the child was feverish from Wednesday till Friday; and the eruptions made their appearance from Friday till Sunday. "They were mostly small, but prominent; and all of them, about twenty in number, went off on the sixth or seventh day. None of them maturated, but some of them exuded a small portion of lymph; which incrusted on the apex, and gave them a warty aspect."

In the Case p. 68, the child was complaining and feverish from Monday till Friday; and from Wednesday till Saturday, eruptions, twenty-five in all, appeared. "The eruption on the pubis had a white, glassy appearance, as if it contained a fluid; but it never became perfectly pustular like other cases. The apices of most of them exuded a small quantity of lymph, which incrusted; and they gradually died away after the seventh day."

In support of his opinion, Mr. Goldson informs us, that several of the most respectable practitioners in his town, among which Messrs. Weymouth, Hill, and Seeds are not omitted, were witness to the above cases, and that they did not hesitate to pronounce them to be "the effect of variolous contagion."

This is all very well; and I shall not contradict these gentlemen. I myself am firmly convinced, that it was the effect of variolous contagion; but it is far from my opinion that they were cases of small-pox, as Mr. Goldson supposes; supposes; and I firmly believe, that under similar circumstances, the same train of symptoms would have been produced in persons who had already undergone the small-pox. There is nothing very extraordinary in this; for I believe it is well proved, that although a person may have had the small-pox, the skin will retain the susceptibility of being infected, when again exposed to the variolous contagion, and a local eruption will take place, frequently attended with fever, and other symptoms of irritation. This eruption has obtained several denominations. The French call it, *petite verole volante;* the Germans, according to the variety of its external characters, have called it *wasser-pocken, wind-pocken, schaaf-pocken, swine-pocken, &c.** the English, in general, have called it the chicken-pox; and sometimes the spurious, or bastard small-pox. In Latin it has been styled varicella. But it signifies little by what name we call it; it is sufficient for us to state, that medical records abound with cases of spurious eruptions, produced by the variolous contagion; which, before the discovery of vaccine inoculation, were frequently mistaken for the small-pox, and looked upon as a second infection; and since that discovery have as frequently, either been considered from ignorance, or misrepresented from a spirit of opposition, as cases of small-pox subsequent to vaccination.

To obviate any rash decisions in future, it will not be amiss to relate a few instances.

Huxham informs us, that he has known several cases of variolous infection in persons who had the small-pox formerly, accompanied with a general eruption very similar to the small-pox, but not attended with fever. He observed this particularly in nurses, and other persons attending upon small-pox patients.

Hensler, formerly a practitioner in Altona, and at present a Professor of the University of Kiel, once a strenuous defender of the small-pox inoculation, at the time when this practice was first introduced into Germany, relates several cases of this description. He mentions, that a child had the small-pox at the same time with an elder sister; that four years afterwards she was infected again by a younger sister, who died of the small-pox. She was feverish for three days; when a general eruption broke out, consisting

* Water-pox, wind-pox, sheep-pox, and swine-pox.

consisting of pustules containing a limpid matter; but they only lasted five days.

Another similar case is that of a woman, who having had the small-pox when young, attended upon one of her children labouring under the confluent disease. She was seized with a violent fever, which lasted twenty-four hours; when six large pustules made their appearance, containing a thin purulent matter. They stood eight days; and did not dry up till a considerable time after.

The following interesting case, related by the same author, shews that the variolous contagion has the power of locally infecting the skin, even when it cannot exert its influence upon the constitution. A lady, who had one of those constitutions which, upon every occasion, had resisted the variolous contagion, and was therefore deemed unsusceptible of the disease, when nursing her child while labouring under the small-pox, was accustomed to make it lean against her cheek; in consequence of which, an eruption of twenty pustules appeared upon her cheek and breast. These pustules disappeared in four days.*

The same is corroborated by Hufeland, who says he has frequently observed, during an epidemic small pox, that when children, who never had the small-pox, slept with others labouring under that disease, instead of being infected with the real small-pox, they were only attacked with a spurious eruption. This eruption, he informs us, was attended with fever, and consisted of pustules containing purulent matter; but as they did not go through their regular course, he did not hesitate to pronounce them spurious; and the event proved he had not been mistaken, for several of these children afterwards caught the real small-pox, and some even during the same epidemic.†

This spurious eruption, proceeding from variolous infection, has been more frequently mistaken for the small-pox than we are aware of. Thus, there are several cases of eruption subsequent to vaccination, to be found in Dr. Woodville's Report, which have been erroneously taken for the small-pox from previous infection; when, in fact, they are nothing more than eruptions of the same nature as

* Hensler Uber die Einempfung der Kenderblatter, 2ter Th. s. 212.

† Bemerkungen Uber die naturlichen und geimpften Blatten von Dr. C. W. Hufeland, 1793, p. 52.

as those above related. The following are the cases I allude to.*

Richard Payne, (Case IV.) tenth day. The pustule was surrounded with a dark inflammatory circle. Fifteenth day, five pustules made their appearance.

William Mundy, (Case XV.) thirteenth day. The pustule was surrounded with an extensive efflorescence in the form of a circle. About the fourteenth day, there appeared several pustules upon his neck and back; they disappeared however in two or three days, without suppuration.

Hannah Hull and Sarah Hull, (Cases XXXI. and XXXII.) In these two sisters, the disease terminated far more favorably than in their brother William Hull; for in both cases, the pustule was surrounded with an efflorescence on the eleventh day; and the number of pustules that appeared in both, was not at all to be compared with those which appeared in their brother; the symptoms which accompanied the eruption in them were likewise of much shorter duration than in their brother.

Maria Murrell, (Case XXXIX.) tenth day. The pustule was surrounded with a diffused efflorescence. Twelfth day, about twenty pustules appeared. Fourteenth day, the pustules appear to dry up.

Richard Colloway, (Case XLIV.) twelfth day. An extensive shining redness surrounded the pustule. At the same time some pustules appeared; their number, however, did not exceed twenty.

Peter Peters, (Case LXXXI.) The efflorescence appeared on the eleventh day; twenty-four pustules appeared, which were all very small.

Sarah Hat, (Case LXXXVI.) eleventh day. The pustule was surrounded with efflorescence. The number of pustules which appeared was about forty.†

The efflorescence which surrounded the pustules in all these cases evidently prove, that no previous infection had taken place; and that the cow-pox had exerted all its influence upon the system. With regard to the subsequent eruptions, it evidently appears from the shortness of their duration,

* Not having a copy of the English original by me, I am obliged to content myself with translating the following extracts from notes taken by me some years ago from a German copy of Dr. Woodville's Reports. It cannot, therefore, be expected, that I shall hit precisely on the same words which Dr. Woodville has made use of.

† Reports of a Series of Inoculations, &c. by William Woodville, M. D. London, 1799.

duration, their smallness in size and number, that it was not the small-pox, although there cannot be the least doubt that they proceeded from exposure to the variolous contagion.

In the same light I look upon the eruption mentioned by Dr. Ballhorn and Mr. Stromeyer, which these gentlemen mistook for a subsequent suppurative vaccine eruption *(eruption vaccine subsequente suppurative)*; for in the cases in which it appeared, there were children in the neighbourhood, and even in the same house, labouring under the small-pox. An antagonist to vaccine inoculation, at Hanover, published in Hufeland's Practical Journal, vol. x. p. 186, one of those cases as an instance of small-pox subsequent to vaccination; and Mr. Ring considers them as cases of small-pox from previous infection.* In justice however to Dr. Ballhorn and Mr. Stromeyer, whatsoever may have been their error, in supposing it to be a suppurative vaccine eruption, I think they have clearly proved, that it was not the small-pox; for they inform us, that when the children had the cow-pox, the pustules on the arms were surrounded by an efflorescence; which could not have taken place had they been previously infected with the small-pox. With regard to the eruption, it resembled the chicken-pox, and was very different from the small-pox, for, "1. The pustules on this eruption were not so broad as in the small-pox. 2. The matter contained in them was more lymphatic than purulent. 3. The number was smaller than is generally the case in small-pox. 4. They had no depression in their apex, as is the case in the small-pox before they suppurate. 5. The scabs, which remained for some time after the pustules dried up, were smaller, thinner, and had a yellow colour; whereas the scabs in the small-pox are of a brownish colour. 6. Some days after they dried up, which generally happened about the sixth or seventh day, hard lumps appeared on the spots which had been covered by the pustules: 7. Which lumps at last went off without leaving the smallest mark behind; and nothing remained but brownish spots, which disappeared after some time."†

It is rather surprizing that Dr. Ballhorn and Mr. Stromeyer should have mistaken the nature of this eruption; for I have no where met with any description, where the distinctions

---

† Traite de l'Inoculation Vaccine, &c. par M. Ballhorn, M.D. Medecin de la Cour, & Mr. Stromeyer, Chirurgien de la Cour a Hanovre, 1801.

* See Treatise on the Cow-pox, &c. by J. Ring, Part II. p. 764 & 765.

distinctions between the chicken-pox and the small-pox are so well marked.

That the cases related by Mr. Goldson are perfectly of the same nature, will appear more evident from the following considerations.

I. This eruption was of a much shorter duration than is observed in the true small-pox. Case II. terminated its course in five days; and the remaining cases in seven days. Thus, about the period that this eruption disappeared, the real small-pox would hardly have begun to maturate.

II. None of them ever maturated, Case III. excepted; but in this case the eruption was too forward; for Mr. Goldson informs us, that as early as the fourth day, several were pustular, and well advanced towards maturation: indeed, they were so far advanced, that in three days after, the whole of them had disappeared. But even maturation would be no proof of its having been the small-pox, for in several of the cases of spurious eruption above related, the pustules contained purulent matter.

III. As these cases never maturated, they never could have passed through the regular stages, so well marked in the small-pox even in its mildest form. About the third or fourth day of the fever, eruptions make their appearance; first, in the form of small red spots, hardly elevated. These gradually increase in size; and after two or three days more, form a small vesicle, which is surrounded by a circular inflamed margin, and contains a clear limpid fluid. For the three or four succeeding days the pustules become larger, more elevated, and acquire their proper figure and size. At the end of this period, the clear limpid fluid contained in the vesicle, is changed into a purulent matter. After a few days more the pustules break, and discharge their matter; which is formed into a crust upon their surface; and lastly, after some time, these crusts fall off, and leave reddish brown spots on the skin below. Had any of Mr. Goldson's cases gone through such a regular course as this, I believe he would have done so much justice to his own cause, as not to have omitted that circumstance.

IV. Neither in Case II. or III. is any thing said, with regard to the external characters of the pustules; but in the cases related in the postscript, Mr. Goldson touches upon this point; and gives us evident proofs, that they were not the small-pox. The eruptions were small, prominent, and had a whitish glassy appearance, as if they contained a fluid; they exuded a small quantity of lymph, which which encrusted on the apex, and gave them a warty appearance. If at the same time we take into consideration, that they never maturated, and only stood seven days; we can no longer doubt but they were cases of genuine varicella.

Perhaps it will be urged, that a fever of three or more days duration occurred in these cases. True, but this does not prove that it was the small-pox. The fever here, was produced by the general irritation; and happens frequently in these spurious eruptions, as we have seen in the cases related by Hensler. Hufeland says, he has seen such cases attended with fever, and a violent delirium; and adds, that even a secondary fever proves nothing; for that it frequently is wanting in the true small-pox, and, on the contrary, is often observed in the spurious, in consequence of irritation.*

The greatest objection, however, made against us will be, that Mr. Goldson took matter from Case III. about the end of the fifth day; and charged four lancets, with which four children were inoculated.

We shall therefore pay particular attention to the result of these inoculations.

Mr. Goldson's own patient was a delicate child, about six months old. It had considerable fever and rash; which was preceded by two or three convulsions; when he could not discover more than eight or ten eruptions, four of which maturated, and all went off on the seventh day.

Of the children inoculated by Messrs. Weymouth and Cooper, one had fifty, the other more than a hundred pustules; these likewise went off in seven days.

The subject inoculated by Mr. Seeds, was a strong and plethoric child at the breast; it had considerable fever, with extensive rash, and more than a thousand pustules; most of which did not turn till the ninth or tenth day.

That morbid matter, whatever may be its nature, when introduced into the human frame, should produce a series of morbid symptoms, is natural to expect. When, therefore, such matter was introduced into the body of a delicate child, only six months old, we need not be surprized at its having fever and convulsions.

With regard to the children inoculated by Messrs. Weymouth and Cooper, no mention is made that they had any fever. If they had any, Mr. Goldson would have mentioned

---

* Hufeland Uber die Blattern, p. 53.

mentioned it; for he is very particular in taking notice of this symptom in his own case, and in that of Mr. Seeds. We therefore must conclude, that these were cases of small-pox, similar to those that once raged in France, in the time of Ettmuller, who very gravely tells us, that the small-pox in France is not accompanied with fever.

The child inoculated by Mr. Seeds had more than a thousand pustules. If these pustules were of the common size of small-pox, this child, who was only eight months old, must have been covered from top to toe; so that I cannot conceive how it was possible for Mr. Goldson to distinguish the extensive rash he talks of. However, in such violent cases of small-pox, there are in general other symptoms attending it, which are not mentioned here; such as, pain at the pit of the stomach upon pressure, swelling of the face, and afterwards of the hands and feet, secondary fever, discharge of saliva, hoarseness, difficulty of swallowing, &c. But we need not wonder at all this; the effect these inoculations produced was as much as could well be expected: the matter was taken from a case of varicella, which terminated its course in seven days, and produced a disease which likewise terminated in seven days, in three cases out of four. That the eruption in the fourth case was protracted two days longer, is only an accidental circumstance, and easily accounted for, the child being strong and plethoric. But independent of this, the number of pustules, and their duration, by no means prove that it was the small-pox. I have frequently witnessed cases of varicella, where the pustules were as numerous, lasted a longer time, and left marks behind like the small-pox. Lest my authority should be deemed insufficient, I shall quote that of an abler judge. Mr. Ring, in his Treatise on the Cow-pox, p. 829, mentions, that many persons have seen the chicken-pox in some measure confluent; and that he himself has known a considerable number leave a cicatrix behind. In p. 835, he relates an instance of a child, in which tne eruption was not complete until the tenth day; and the last vesicle did not disappear till the fourteenth.

These last inoculations, therefore, with matter taken from Case 3d. instead of removing our doubts, only tend to increase them; and give us convincing proofs of Mr. Goldson's mistaken notions. Should he persist in them, and reason be now insufficient, time will probably convince him of his errors when it is too late, when the unfortunate subjects of these experiments will be snatched

 away away from their deluded parents, and fall victims to that dire disease the small-pox.

Should this indeed unfortunately take place, it would not be the first time that thousands have been deceived in this respect, from having paid too little attention to the distinctions between the variola and varicella: and when we consult medical records, we too often meet with blunders, which reflect little honour on the profession. Mr. Ring has related several cases of this description; and if Mr. Goldson had consulted them, perhaps he would have been rather more circumspect in making experiments. Among others, there are two cases communicated by Mr. Paytherus, where an elderly surgeon inoculated his grand-daughter with supposed variolous matter; "and showed the case to his friends, as one of very mild small-pox. Mr. Paytherus having expressed his doubts whether the disorder was the small-pox, gave great offence. About three years after, the young lady had the small-pox of the confluent kind."

"This man of Ross pronounced another case of chicken-pox, concerning which he was consulted, to be a case of small-pox. Mr. Paytherus informed the parents, it was nothing but the chicken-pox; and, inoculating the patient soon afterwards with proper matter, produced the small-pox."

Mr. Ring adds another melancholy instance, which occurred in town some years ago. "A lady, who was going abroad, had her daughter inoculated by one of those impostors who, having swept out a drug-shop for some years, called himself an apothecary. When the young lady returned to England, she caught the small-pox, and died."*

Dr. Lettsom, in his observations on the cow-pox, as quoted by Mr. Ring, observes, that he had lately attended two young persons under the small-pox, each an only child of considerable family, who had been inoculated two or three years before by respectable men; and the mothers of the children shewed him, "what they conceived to be the marks, or pitting, from the inoculated small-pox. Happily they both recovered from an alarming eruption of the disease;" but, he adds, "Two relations I once claimed, who were inoculated with matter supposed to be variolous, by an eminent inoculator, afterwards caught the small-pox,

* Ring's Treatise, &c. Part II. p. 333.

pox, and to one of them it proved fatal."* I could add several other cases, which have happened in our times.

But we need not confine ourselves to our own times; we find such cases in older authors. Monro the grandfather, in his history of the small-pox inoculation in Scotland, says, that he knows several instances of the bastard kinds of small-pox, as he calls them, having been mistaken for the true small-pox. Both Cullen and Heberden maintain that medical men were often deceived by the chicken-pox, from its assuming the appearance of the small-pox. Fritze, in his Medical Annals, says, he has seen so great a similarity between the chicken-pox and small-pox, that he had very nearly been led into a mistake, and taken matter from them. Bond, in his defence of the small-pox inoculation, maintains, that if proper attention had been paid to the difference between the true and spurious small-pox, it would have saved the lives of thousands. Elsner, in his remarks upon the small-pox inoculation, cautions us to be on our guard not to mistake for the real small-pox, that species of spurious small-pox, which suppurates and can be propagated by inoculation; but which will by no means prevent an attack of the true small-pox. Condamine maintained, that the distinctions of eruptive diseases were far from being ascertained. Tralles made no scruple to say, that he believed a physician might be deceived, and take the chicken-pox for the small-pox. Schultz relates an anecdote of Gaubius, that once an eruptive disease was shown to him, which he declared not to be the small-pox; upon which another person reproached him that he did not know what the small-pox was. The event however proved, that Gaubius was not mistaken; as the patient afterwards caught the real disease.

Cases, therefore, are not wanting to prove, how often errors of this sort have been committed; and it is not to be wondered at in the least, that from such a want of knowledge, the idea should have arisen of persons having the small-pox twice. There are other cases on record, which have led to the same erroneous opinion; and it will perhaps not be amiss to consider some of them here. The first I shall take notice of, are cases of spurious eruptions, produced by inoculations with matter from the true small-pox.

* Ring, p. 860.

Dr. Jenner, in his first Treatise, relates an instance of a gentleman, who used to carry about him variolous matter, received on lint or cotton, and put into a phial while in a fluid state. The warmth of his pocket produced putrefaction. Hence, although inflammation, swelling of the axillary glands, fever, and sometimes eruptions, were excited, yet the unfortunate patients were as subject to the small-pox as before; and many, who thought themselves in perfect security, fell victims to that horrible disease.

Dr. Jenner mentions other cases, which occurred to Mr. Earle, surgeon, at Frampton upon Severn. This gentleman took matter from a pustule too far advanced, and inoculated five persons with it. In all, the arms inflamed, with fever, and swelling in the axilla. About the ninth day, an eruption appeared; which, however, dried away sooner than usual. Four of these persons afterwards caught the small-pox in the natural way, one of whom died, three recovered, and the other, being cautioned by Mr. Earle to avoid as much as possible the chance of catching it, escaped the disease through life.

A similar circumstance again occurred to this gentleman. He inoculated three children with matter procured by another person. The arms inflamed properly; fever and pain in the axilla came on; and in ten days eruptions appeared, which disappeared in two days. Being somewhat alarmed for the safety of these patients, from a similarity of their cases to those already mentioned, he inoculated them a second time with matter in its most perfect state; in consequence of which, they all took the infection of the small-pox again, and all had a full burthen.

Similar cases occurred to Bond, in his own practice. He inoculated several children with matter, which, as afterwards appeared, had undergone a decomposition. The children all had a spurious disease. He inoculated them afterwards with proper matter, and they had the true small-pox.

In all these cases, the matter was either taken at too late a period, or had suffered a decomposition; but there are cases related, where matter in its most perfect state was used, and still produced a spurious small-pox. Thus Bond mentions, that at one time he inoculated five persons with fresh matter, and all five got a spurious small-pox. At another time, he saturated a thread with variolous matter, and inoculated several persons with it, all of whom had the true small-pox. Part, however, of the same thread hread he sent to his brother, who inoculated twelve persons with it, and all had a spurious small-pox.*

Elsner observes, that some experiments prove that matter taken from the true small-pox, when inserted into the body of some subjects, undergoes such changes as to produce an eruption, which pursues the course of the spurious small-pox; but that, in these cases, such an eruption does not prevent an attack of the true small-pox. He adds the following observation. Whatever is observed in the artificial infection by inoculation, holds true in the natural infection; and the variolous matter, under certain circumstances, may be so much weakened, as to produce a spurious eruption instead of the true small-pox.†

Vogel says, it is certain that, after the inoculation with the true small-pox matter, sometimes a species of spurious small-pox is produced, which is no preventive of the recurrence of the disease. He adds, this proves that the system is not always prepared to produce the true small-pox.‡

Cüsson asserts the same, and relates an instance where he saw two children inoculated with the most genuine small-pox matter. The arms inflamed in the usual manner, attended with fever, vomiting, &c. on the seventh day, which was followed by an eruption that did not maturate. The gentleman who had performed the inoculation, was perfectly satisfied that the children had undergone the true disease; the more so, as he had taken matter from the arms of these children, and inoculated others with it successfully. The event, however, proved that he was mistaken; for these children afterwards caught the small-pox in the natural way.||

The last cases I shall consider, which have led to the idea of a second infection of the small-pox, are either those in which, from improper treatment, an imperfect eruption has taken place, or where, by a supervening disease, the small-pox has been for a while suspended. In the first instance, the matter retained will afterwards find its way outwards, sometimes in the form of abscesses, but most frequently in that of an eruption perfectly similar to the

* Vertheidegung der Einpfropfung der Blattern, s. 63.

† Ein Paar Worte Uber die Inoculation, s. 47.

‡ Handbuch, &c. Th. 3, s. 10.

|| Cüsson, Recherches sur les irregularités, qui presente quelque fois dans sa marche la petite verole inoculée, et sur la confiance, qui menteut ces sortes d'inoculations irregulaires.

the real small-pox. In the latter, the small-pox will reappear, and finish its regular course as soon as the supervening disease has ceased to exert its influence upon the system.

Sidobre relates an instance of a lady of quality, who, after such an imperfect eruption of the small-pox, was not only attacked with fistulous sores, but affected, during the period of eighteen months, with an eruption similar to the small-pox; and a fresh crop of pustules used to break out again, as soon as the former had fallen off.

De Haen mentions, that a boy, three years old, after a severe and ill treated small-pox, continued to be in a weak state of health; that, fortunately, an abscess was formed on his breast, which for some months continued to discharge a quantity of matter. After this, the child began to recover; and in a short space of time, an eruption perfectly similar to the small-pox, again made its appearance.

Hensler corroborates the same; he mentions, that a child had the small-pox in so mild a form, as to require no confinement. He saw the child three weeks after: the pustules were then dried up; the crust, however, did not fall off, but adhered to the skin below, and discharged a sanies which excoriated the parts immediately surrounding it. They itched considerably; and the child was fretful. Hensler ordered the parents to give him from time to time a mild laxative; nevertheless, four days after, the child sickened again, and an eruption appeared, which in some places was confluent. After the eruption dried up, this unfortunate sufferer died of pneumonia.

This author likewise relates, that two boys caught the infection from an elder brother, who laboured under the small-pox. The eldest became feverish on the 3d of January 1762, and continued so till the 10th, when small red spots made their appearance. On the 11th, they were elevated. On the 14th, they were filled with matter; and about the 16th, they began to dry up; the scabs, however, remained for a long while after. Since that time, the boy continued more or less sickly till the beginning of April, when he became feverish again; and after two days an eruption appeared, consisting of large pustules, surrounded by little or no inflammation. They were broad and flat, and filled with a yellow purulent matter. They dried up very slowly. In the youngest the fever took place at the same time with that of the eldest; but he had fewer eruptions. When these dried up, a new crop succeeded; and this succession of of fresh crops coming, and going again, continued till April; when the last and most copious eruption took place.

Hufeland relates several instances of the same description. He is of opinion that the antiphlogistic regimen, carried into extremes, too great exposure to cold air, and the abuse of mercurial purgatives, have been the causes which have produced them. He has once seen it occasioned by a strong emetic, given at the beginning of the disease. He likewise mentions several instances, where the measles, the scarlet fever, &c. had suspended the small-pox for several days; and two cases of inoculated small-pox, where the arms properly inflamed, accompanied by fever, and eruptions of small red spots, together with a strong variolous smell; yet a stop was put to the farther progress of the disease by a supervening influenza. These children, eight weeks afterwards, caught the small-pox in the natural way.*

From a due consideration of the above facts, I must confess that I am rather sceptically inclined, when cases are related, of persons having the small-pox twice. It would be presumptuous in me to deny the possibility of a second infection; several of the most eminent men in the profession have admitted it; and it is with deference to their superior judgment, that I wish to offer mine.

All that I mean to demonstrate is, that the study of this important subject has been shamefully neglected; and that the distinctions between the true and the spurious small-pox are but imperfectly known; witness the pamphlet under consideration. When, therefore, we hear of such cases, we ought to be very cautious, before we pronounce our opinion.

The idea, however, of a second infection of the small-pox, is by no means of modern origin. It seems to have been a favorite topic among the older medical writers. *Amatus* relates, that children, and even adults, who had the small-pox, were infected again at Ancona, in the year 1551. *Forestus* mentions, that at Delft, in the year 1562, and 1563, during a foggy season, not only children and adults, but likewise some old people, who had undergone the small-pox and the measles, were again attacked with similar eruptive diseases. Stalpart Vander Wiel saw a child who, three weeks after the small-pox, was infected a second time. Diemerbroek, during a violent epidemic

* Hufeland Uber die Blattern, p. 262, 263.

mic, frequently saw a second attack of the small-pox, when the patients were scarcely recovered from the first. Some had, in the course of six months, three different eruptions. A son of Forestus, four years old, had, in the year 1551, the small-pox twice; and afterwards, the measles. Similar instances are said to have occurred in the provinces of Holland, Zealand, and Guelderland, during the epidemic in the summer of 1753. Something more wonderful than all this is related by Forestus. He tells us, that a certain good woman at Boulogne had the small-pox seven times, and died of it in the 118th year of her age.

Ere I conclude, it is incumbent on me to state, that I have made inquiries relative to Mr. Goldson's assertion, that cases of failures have happened in the Isle of Wight. The result of them is, that a child, under the care of a medical practitioner at Newport, had the chicken-pox after the cow-pock; and an enemy to vaccination propagated a report that it was the small-pox. Another case occurred to Mr. Morton, of the Hospital Staff; he himself informed me, that he was under the necessity of taking matter from a pustule too far advanced; that the child inoculated with it had a spurious cow-pock; and that it caught the small-pox before he had an opportunity of inoculating it again. Mr. Goldson pretends to forbear noticing any cases upon mere report; yet he takes notice of them whenever he can; and since his publication, he and his friends have circulated such reports with great industry.

In taking leave of Mr. Goldson, I earnestly advise him to abandon the weapons he has hitherto employed. Idle tales are of little avail; and, even by his friends, can only be looked upon as a last resource.

He who, from a zeal for the discovery, should suffer his eyes to be shut against conviction, and attempt to conceal its failures, would indeed commit an act beneath the dignity of the profession; but he who imposes on the ignorant, under the mask of candour and moderation; who spreads vain alarms, and provokes controversy upon a subject in which he must so sensibly feel his own deficiency, is guilty of a deed far more beneath the dignity of the profession; and far more unbecoming one, who is intrusted with the happiness and welfare of the public.

*Isle of Wight, August* 27, 1804.

