# Errata

**Published:** 1804-11-01

**Authors:** 


### ERRATA.

P. 305, l. 6, from the bottom, dele the words "but once."

324, l. 4, place a semicolon after "time;" and dele the pronoun "that."

